# Differential liver function at cessation of atezolizumab-bevacizumab versus lenvatinib in HCC: a multicenter, propensity-score matched comparative study

**DOI:** 10.3389/fonc.2024.1372007

**Published:** 2024-02-28

**Authors:** Ji Won Han, Pil Soo Sung, Jae-Sung Yoo, Hee Sun Cho, Soon Kyu Lee, Hyun Yang, Ji Hoon Kim, Heechul Nam, Hae Lim Lee, Hee Yeon Kim, Sung Won Lee, Do Seon Song, Myeong Jun Song, Jung Hyun Kwon, Chang Wook Kim, Si Hyun Bae, Jeong Won Jang, Jong Young Choi, Seung Kew Yoon

**Affiliations:** ^1^ The Catholic University Liver Research Center, College of Medicine, The Catholic University of Korea, Seoul, Republic of Korea; ^2^ Division of Gastroenterology and Hepatology, Department of Internal Medicine, College of Medicine, Seoul St. Mary’s Hospital, The Catholic University of Korea, Seoul, Republic of Korea; ^3^ Division of Gastroenterology and Hepatology, Department of Internal Medicine, College of Medicine, Incheon St. Mary’s Hospital, The Catholic University of Korea, Incheon, Republic of Korea; ^4^ Division of Gastroenterology and Hepatology, Department of Internal Medicine, College of Medicine, Eunpyeong St. Mary’s Hospital, The Catholic University of Korea, Seoul, Republic of Korea; ^5^ Division of Gastroenterology and Hepatology, Department of Internal Medicine, College of Medicine, Uijeongbu St. Mary’s Hospital, The Catholic University of Korea, Uijeongbu, Republic of Korea; ^6^ Division of Gastroenterology and Hepatology, Department of Internal Medicine, College of Medicine, Bucheon St. Mary’s Hospital, The Catholic University of Korea, Bucheon, Republic of Korea; ^7^ Division of Gastroenterology and Hepatology, Department of Internal Medicine, St. Vincent’s Hospital, College of Medicine, The Catholic University of Korea, Suwon, Republic of Korea; ^8^ Division of Gastroenterology and Hepatology, Department of Internal Medicine, Daejeon St. Mary’s Hospital, College of Medicine, The Catholic University of Korea, Daejeon, Republic of Korea

**Keywords:** HCC, atezolizumab plus bevacizumab, lenvatinib, residual liver function, survival

## Abstract

**Background:**

Atezolizumab+bevacizumab (AB) and lenvatinib have been proposed as first-line treatment options for patients with advanced hepatocellular carcinoma (HCC), but comparative efficacy and associated factors are controversial.

**Materials and methods:**

This real-world multicenter study analysed patients with HCC who received AB (n=169) or lenvatinib (n=177).

**Results:**

First, 1:1 propensity score matching (PSM) was performed, resulting in 141 patients in both the AB and lenvatinib groups. After PSM, overall survival (OS) was better in the AB group than in the lenvatinib group [hazard ratio (HR)=0.642, P=0.009], but progression-free survival (PFS) did not vary between the two groups (HR=0.817, P=0.132). Objective response rate (ORR) was also similar between AB and lenvatinib (34.8% vs. 30.8%, P=0.581). In a subgroup of patients with objective responses (OR, n=78), OS (HR=0.364, P=0.012) and PFS (HR=0.536, P=0.019) were better in the AB group (n=41) than in the lenvatinib group (n=37). Time-to-progression from time of OR was also better in the AB group (HR=0.465, P=0.012). Importantly, residual liver function was a significant factor related to OS in both treatments. Child-Pugh score following cessation of the respective treatments was better in the AB group (n=105) than in the lenvatinib group (n=126) (median 6 versus 7, P=0.008), and proportion of salvage treatment was also higher in the AB group (52.4% versus 38.9%, P=0.047). When we adjusted for residual liver function or salvage treatment, there was no difference in OS between the two treatments.

**Conclusion:**

Our study suggests that residual liver function and subsequent salvage treatments are major determinants of clinical outcomes in patients treated with AB and lenvatinib; these factors should be considered in future comparative studies.

## 1 Introduction

The REFLECT and IMbrave150 trials have shown that lenvatinib and atezolizumab plus bevacizumab (AB) have better clinical outcomes than sorafenib in advanced, unresectable hepatocellular carcinoma (HCC) (1, 2). In the REFLECT trial, lenvatinib showed comparable overall survival (OS) compared to sorafenib (median 13.6 versus 12.3 months), whereas it had better progression-free survival (PFS) (median 7.4 versus 3.7 months, P<0.001) and objective response rate (ORR) (24.1% versus 9.2%, P<0.001) (1). In the IMbrave150 trial, AB had superior OS (19.2 versus 13.4 months, P<0.001), PFS (6.9 versus 4.3 months, P<0.001), and ORR (30.0% versus 11.9%, P<0.001) to lenvatinib (2).

Consequently, these therapeutic regimens have been endorsed as first-line treatment options for patients with advanced HCC. Notwithstanding these recommendations, a prevailing debate exists concerning which of the two is the most optimal for first-line treatment. Some investigations posit that AB is superior to lenvatinib in terms of OS (3–5), whereas alternative studies assert the contrary (6, 7). Moreover, some reports indicated no significant difference in efficacy between the two regimens (8–10).

Liver function, tumor size, tumor extension into adjacent structures, patient performance status, and extrahepatic metastases serve as pivotal prognostic indicators in individuals diagnosed with HCC (11). Additionally, hepatitis B or C infections, as well as serum concentrations of tumor markers—namely alpha-fetoprotein (AFP) and protein induced by vitamin K absence or antagonist-II (PIVKA-II)—should be taken into account when assessing prognostic factors. Furthermore, subsequent treatment can extend survival in patients who discontinue first-line therapy due to tumor progression or adverse events (AEs) whose performance status and liver function are adequate to tolerate further treatment (12), suggesting that the capability of patients to undergo second-line therapy following the termination of initial treatment stands as a significant factor closely associated with clinical outcomes.

However, previous studies comparing these two treatments did not take into account various prognostic factors including residual liver function and salvage treatment, nor did they conduct subgroup analyses based on these factors. In this real-world, multi-center study utilizing propensity-score matching (PSM), we conducted comparative analyses between lenvatinib and AB in terms of OS, PFS, and ORR. Of note, we conducted subgroup analyses that considered baseline factors, as well as residual function and salvage treatments, to determine which factors influence the differences in clinical outcomes between the two treatments.

## 2 Materials and methods

### 2.1 Study cohort

Ethical approval for this research was granted by the Institutional Review Board of the Catholic University of Korea (approved number: XC23RADI0081), and the investigation was conducted in adherence to the principles delineated in the Declaration of Helsinki. A retrospective analysis was undertaken on 346 consecutive patients with unresectable HCC who were treated with AB or lenvatinib at seven affiliated hospitals in Korea. For the AB arm, patients were enrolled between September 2020 and December 2022; for the lenvatinib arm, the enrollment period was from January 2019 to December 2022. Diagnoses of HCC were confirmed either histologically or through radiological examinations, specifically contrast-enhanced computed tomography (CT) and/or magnetic resonance imaging (MRI). Inclusion criteria comprised: (1) histologically or radiologically confirmed intermediate to advanced HCC not amenable to surgical resection; (2) minimum age of 18 years; and (3) an Eastern Cooperative Oncology Group (ECOG) performance status score not exceeding 2. Exclusion criteria included: (1) absence of follow-up post-initiation of therapy; (2) less than a two-week treatment course with lenvatinib; (3) fewer than two cycles of AB; and (4) prior malignancies other than HCC within the past five years.

### 2.2 Therapeutic protocol

Lenvatinib dosing was stratified by patient body weight: 8 mg daily for those weighing less than 60 kg and 12 mg daily for those above 60 kg. The AB therapeutic regimen consisted of intravenous administration of 1200 mg atezolizumab in conjunction with 15 mg/kg bevacizumab, repeated tri-weekly until either disease progression or onset of unacceptable toxicity.

### 2.3 Efficacy and adverse event assessment

Patients were stratified by the Barcelona Clinic Liver Cancer (BCLC) stage, utilizing radiological and laboratory data at the time of enrollment. Periodic imaging, via CT or MRI, was scheduled at 4-12 weeks intervals for lenvatinib and every 3-4 cycles for AB to evaluate treatment responses based on the modified Response Evaluation Criteria in Solid Tumors (mRECIST), as previously delineated (13). OS and PFS were measured from treatment initiation to the date of death, last follow-up, or disease progression. ORR was calculated as the sum of the “complete” and “partial” responses at the response evaluation. The disease control rate (DCR) was calculated as the sum of the complete response (CR), partial response (PR), and stable disease (SD). Additionally, the modified albumin-bilirubin (mALBI) score was quantified to gauge hepatic function using a predetermined formula (14). AEs were characterized according to the Common Terminology Criteria for Adverse Events version 4.0 (15).

### 2.4 Statistical methods

Statistical computations were conducted employing R statistical software (version 4.0.3; R Foundation Inc., Vienna, Austria; http://cran.r-project.org, accessed on 6 September 2021) and SPSS version 23.0 software (IBM Corp., Armonk, NY, USA). Continuous variables were compared via Student’s t-test, and categorical variables were compared via chi-square test. To counterbalance baseline differences between the AB (n=169) and lenvatinib (n=177) cohorts, PSM was applied using one-to-one nearest-neighbor matching within a 0.20 caliper width, resulting in 141 patients in each matched group. Kaplan-Meier estimations were employed for survival analyses, and Cox regression modeling was utilized for survival outcome determinants. Statistical significance was established at p-values < 0.05.

## 3 Results

### 3.1 Baseline characteristics

**Table 1** shows the comparison of baseline characteristics between AB (n=169) and lenvatinib (n=177) groups before and after PSM. Before PSM, demographic characteristics including gender, age, and etiology were comparable between the two groups. Additive combined treatment on each regimen was comparably performed in the two groups. However, the proportion of treatment-naïve patients was significantly higher in the AB group (60/169, 35.5% versus 36/177, 20.3%, P=0.002). Regarding laboratory tests, the serum levels of aspartate aminotransferase (AST), alanine aminotransferase (ALT), albumin, platelets, international normalized ratio (INR), and creatinine as well as tumor markers such as AFP and PIVKA-II showed no significant differences between the two groups. However, the total bilirubin level was higher in the lenvatinib group (mean 1.2 versus 1.0 mg/dL, P=0.009). Furthermore, ascites, Child-Pugh class, and ECOG were not different between the two groups. Tumor factors including largest intrahepatic tumor size, multiple intrahepatic tumors, portal vein invasion (PVI), and extrahepatic spread were comparable, and mUICC and BCLC stages were also not different between the two groups. PSM was performed, and there was no difference in the baseline characteristics between the two groups. All subsequent analyses were performed using the matched cohort unless stated that an unmatched cohort was used.

**Table 1:** Comparison of baseline characteristics between lenvatinib and atezolizumab+bevacizumab groups before and after propensity-score matching.

	Before matching		P	After matching		P
	Lenvatinib n=177	AB n=169		Lenvatinib n=141	AB n=141	
Male gender	152 (85.9)	144 (85.2)	0.981	118 (83.7)	118 (83.7)	1.000
Age, years	63.6 ± 11.8	63.8 ± 11.5	0.906	63.8 ± 11.8	63.6 ± 11.2	0.852
Treatment naïve	36 (20.3)	60 (35.5)	0.002	36 (25.5)	34 (24.1)	0.890
Viral etiology	127 (71.8)	113 (66.9)	0.385	100 (70.9)	97 (68.8)	0.795
HBV	109 (61.6)	102 (60.4)	0.902	86 (61.0)	87 (61.7)	1.000
HCV	18 (10.2)	12 (7.1)	0.411	14 (9.9)	11 (7.8)	0.675
Alcohol	47 (26.6)	45 (26.6)	1.000	41 (29.1)	34 (24.1)	0.419
Others	20 (11.3)	25 (14.8)	0.420	16 (11.3)	22 (15.6)	0.383
Combined treatment	28 (15.8)	24 (14.2)	0.787	21 (14.9)	20 (14.2)	1.000
Radiotherapy	24 (13.6)	20 (11.8)	0.749	17 (12.1)	16 (11.3)	1.000
TACE	4 (2.3)	0 (0.0)	0.144	4 (2.8)	0 (0.0)	0.131
Surgery	0 (0.0)	3 (1.8)	0.230	0 (0.0)	3 (2.1)	0.246
Laboratory tests						
AST, U/L	86.4 ± 99.1	79.7 ± 81.9	0.492	78.9 ± 82.0	76.4 ± 85.5	0.801
ALT, U/L	44.4 ± 62.3	37.5 ± 34.3	0.201	39.8 ± 57.8	35.5 ± 35.5	0.448
Total bilirubin, mg/dL	1.2 ± 0.8	1.0 ± 0.7	0.009	1.1 ± 0.7	1.0 ± 0.7	0.298
Albumin, g/dL	3.7 ± 0.5	3.8 ± 0.5	0.818	3.7 ± 0.5	3.8 ± 0.4	0.594
Platelet, 10^9^/L	167.0 ± 98.0	179.9 ± 93.7	0.213	173.2 ± 98.9	173.1 ± 90.7	0.990
INR	2.2 ± 13.7	1.1 ± 0.1	0.299	1.1 ± 0.1	1.1 ± 0.1	0.305
Creatinine, mg/dL	0.9 ± 0.7	0.9 ± 0.6	0.802	0.8 ± 0.3	0.9 ± 0.6	0.577
AFP, ng/mL	29673.3 ± 251757.9	12036.0 ± 33419.1	0.357	34560.4 ± 281596.2	12854.0 ± 35836.0	0.365
PIVKA-II, mAU/mL	22275.3 ± 59208.0	21379.1 ± 58625.5	0.888	21021.3 ± 59381.1	20959.3 ± 62518.5	0.993
Ascites	35 (19.8)	33 (19.5)	1.000	30 (21.3)	27 (19.1)	0.767
Child-Pugh class B	29 (16.4)	18 (10.7)	0.209	24 (17.0)	15 (10.6)	0.168
ECOG			0.212			0.229
1	63 (35.6)	55 (32.5)		48 (34.0)	43 (30.5)	
2	1 (0.6)	5 (3.0)		1 (0.7)	5 (3.5)	
Largest intrahepatic tumor size	7.1 ± 7.4	7.7 ± 5.5	0.376	7.3 ± 7.8	7.1 ± 5.5	0.809
Multiple intrahepatic tumor	131 (74.0)	121 (71.6)	0.701	102 (72.3)	98 (69.5)	0.694
Portal vein invasion	96 (54.2)	85 (50.3)	0.531	69 (48.9)	68 (48.2)	1.000
mUICC stage			0.644			0.405
2	9 (5.1)	5 (3.0)		7 (5.0)	5 (3.5)	
3	20 (11.3)	15 (8.9)		18 (12.8)	10 (7.1)	
4A	57 (32.2)	53 (31.4)		42 (29.8)	42 (29.8)	
4B	91 (51.4)	96 (56.8)		74 (52.5)	84 (59.6)	
BCLC stage			0.480			0.394
B	23 (13.0)	17 (10.1)		20 (14.9)	15 (10.6)	
C	154 (87.0)	152 (89.9)		120 (85.1)	126 (89.4)	
Extrahepatic spread	106 (59.9)	103 (60.9)	0.927	87 (61.7)	90 (63.8)	0.805
Lung	64 (36.2)	62 (36.7)	1.000	51 (36.2)	53 (37.6)	0.902
Lymph node	36 (20.3)	28 (16.6)	0.445	32 (22.7)	26 (18.4)	0.461
Adrenal	5 (2.8)	6 (3.6)	0.938	3 (2.1)	6 (4.3)	0.498
Bone	16 (9.0)	23 (13.6)	0.241	14 (9.9)	19 (13.5)	0.459
Peritoneal seeding	7 (4.0)	8 (4.7)	0.927	5 (3.5)	8 (5.7)	0.570

Data are given as n (%) or mean ± standard deviation. AB, Atezolizumab plus Bevacizumab; HBV, Hepatitis B Virus; HCV, Hepatitis C Virus; TACE, Transarterial Chemoembolization; AST, Aspartate Aminotransferase; ALT, Alanine Aminotransferase; INR, International Normalized Ratio; AFP, Alpha-fetoprotein; PIVKA-II, Protein Induced by Vitamin K Absence or Antagonist-II; ECOG, Eastern Cooperative Oncology Group; mUICC, Modified Union for International Cancer Control; BCLC, Barcelona Clinic Liver Cancer.

### 3.2 Comparison of clinical outcomes

**Figure 1:**
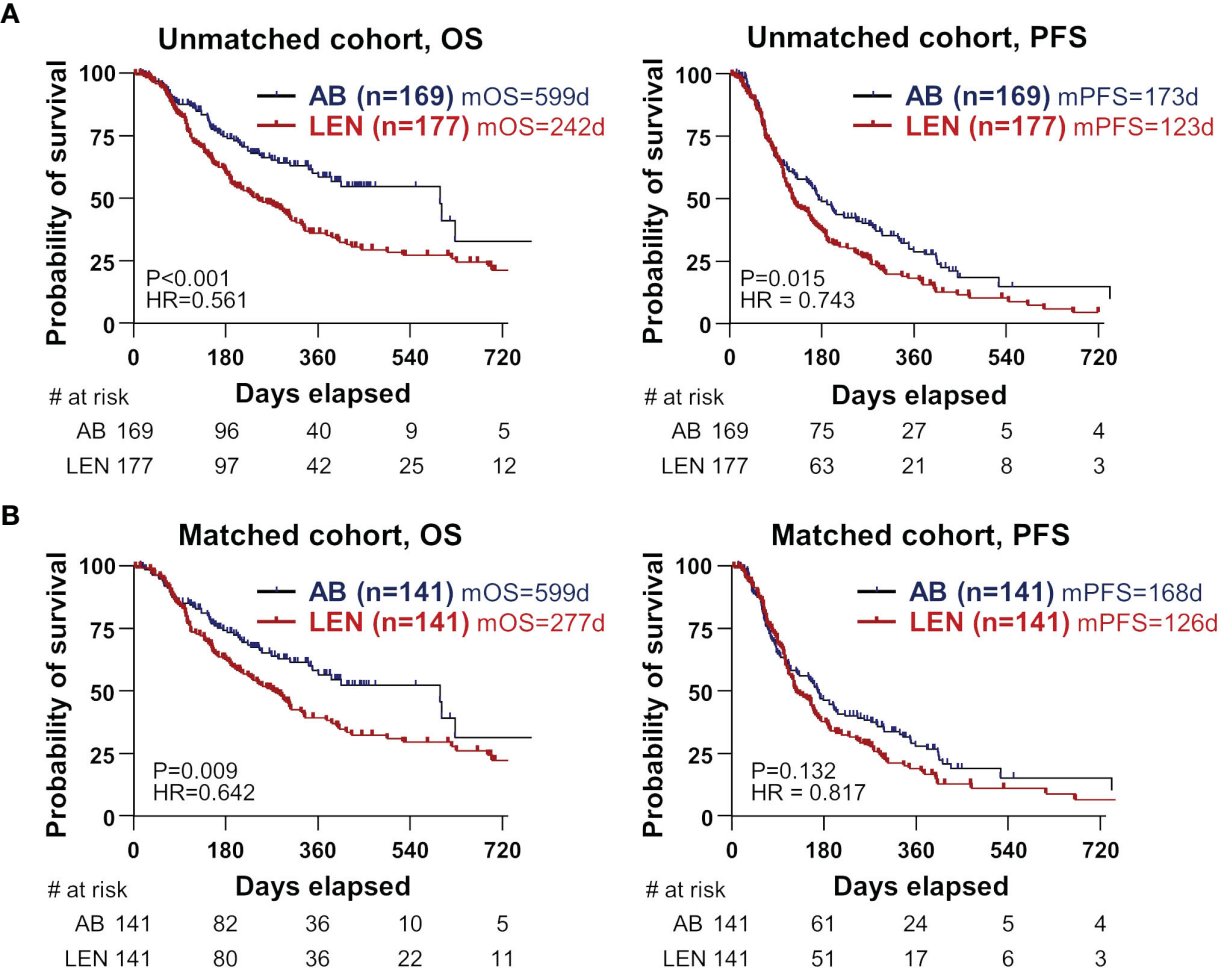
Survival differences between the two treatments in unmatched and matched cohorts. **(A, B)** OS and PFS were compared between AB- and LEN-treated patients, before **(B)** and after propensity-score matching. OS, overall survival; PFS, progression-free survival; AB, atezolizumab+bevacizumab; LEN, lenvatinib; m, median; d, days; HR, hazard ratio.

We investigated whether there are differences in clinical outcomes such as OS, PFS, ORR, and DCR between the two treatments. In the unmatched cohort, AB showed better OS and PFS compared to lenvatinib (**Figure 1A**). In the matched cohort, AB also showed significantly better OS than lenvatinib (median 599 versus 277 days, HR=0.642, P=0.009) (**Figure 1B**). However, PFS was not different between the two groups (median 168 days for AB, versus 126 days for lenvatinib, HR=0.817, P=0.132) (**Figure 1B**). Before and after PSM, ORR and DCR were not significantly different between the two groups (**Table 2**). In the matched cohort, ORR was 30.8% (37/120) in the lenvatinib group and 34.8% (41/118) in the AB group (P=0.581). Furthermore, DCR was 70.0% (84/120) in the lenvatinib group and 75.4% (89/118) in the AB group (P=0.384).

**Table 2:** Best responses of each treatment before and after matching.

	Before matching		P	After matching		P
	Lenvatinib n=177	AB n=169		Lenvatinib n=141	AB n=141	
CR	7 (4.0)	7 (4.1)		6 (4.3)	7 (5.0)	
PR	37 (20.9)	42 (24.9)		31 (22.0)	34 (24.1)	
SD	56 (31.6)	57 (33.7)		47 (33.3)	48 (34.0)	
PD	49 (27.7)	36 (21.3)		36 (25.5)	29 (20.6)	
undetermined	28 (15.8)	27 (16.0)		21 (14.9)	23 (16.3)	
ORR	44 (29.5)	49 (34.5)	0.381	37 (30.8)	41 (34.8)	0.581
DCR	100 (67.1)	106 (74.7)	0.197	84 (70.0)	89 (75.4)	0.384

Data are given as n (%). AB, Atezolizumab plus Bevacizumab; CR, Complete Response; PR, Partial Response; SD, Stable Disease; PD, Progressive Disease; ORR, Objective Response Rate; DCR, Disease Control Rate.

Next, we investigated which treatment would be beneficial in each subgroup in terms of OS and PFS. As a result, in the respective patient subgroups of age >65 years, viral etiology, ALBI grade 1, AFP>1000 ng/mL, PIVKA-II>1000 mAU/mL, ECOG 0, Child-Pugh 5A, largest intrahepatic tumor >5 cm, multiple intrahepatic tumors, or PVI, AB had significant benefits in OS (**Supplementary Table 1**). In addition, patient subgroups of ALBI grade 1, PIVKA-II>1000, largest intrahepatic tumor >5 cm, multiple intrahepatic tumors, or PVI also showed benefits in PFS from AB compared to lenvatinib (**Supplementary Table 2**).

### 3.3 Factors associated with the clinical outcomes

We subsequently analysed factors associated with the OS (**Table 3**), PFS (**Supplementary Table 3**), and objective response (OR) (**Supplementary Table 4**) in the total, lenvatinib, and AB groups using multivariate analyses. Among the lenvatinib subgroup, high AST level, low albumin level, multiple intrahepatic tumors, high mUICC stage, and poor residual liver function after cessation of treatment were factors associated with poor OS. In the AB subgroup, high AST level, low albumin level, poor ECOG, and poor residual liver function were associated with poor OS, whereas multiple intrahepatic tumors or high mUICC stage were not significant. In terms of PFS, high AST level, poor baseline Child-Pugh score (CPS), multiple intrahepatic tumors, and high mUICC stage were related to poor PFS in the lenvatinib group, whereas low albumin level, ascites, poor ECOG, and high mUICC stage were significant in the AB group. Regarding OR, AST and ECOG were associated factors only in the total cohort, but not in the two treatment subgroups.

**Table 3:** Multivariate Cox-regression analyses* of factors associated with overall survival in the matched cohort.

	Total (n=282)		Lenvatinib (n=141)		AB (n=141)	
	HR	P	HR	P	HR	P
AST	1.002 (1.00-1.00)	0.087	1.005 (1.00-1.01)	0.002	1.005 (1.00-1.01)	0.022
Albumin	0.546 (0.37-0.81)	0.003	0.524 (0.33-0.83)	0.006	0.174 (0.06-0.48)	<0.001
ECOG	1.799 (1.28-2.52)	0.001	1.104 (0.65-1.88)	0.716	2.946 (1.64-5.30)	<0.001
Multiple intrahepatic tumor	1.975 (1.24-3.15)	0.004	3.065 (1.63-5.74)	0.001	1.213 (0.49-2.96)	0.672
Portal vein invasion	1.467 (1.00-2.15)	0.049	1.212 (0.67-2.20)	0.529	1.594 (0.66-3.83)	0.297
mUICC stage	not included		1.728 (1.30-2.30)	0.001	not included	
Residual liver function**	1.241 (1.14-1.36)	<0.001	1.304 (1.16-1.47)	<0.001	1.252 (1.08-1.46)	0.004

AB, Atezolizumab plus Bevacizumab; HR, hazard ratio; AST, Aspartate Aminotransferase; ECOG, Eastern Cooperative Oncology Group; mUICC, Modified Union for International Cancer Control.

*Only factors with P<0.02 in univariate analyses were included for multivariate analyses.

**Child-Pugh score at the time of cessation of lenvatinib or Atezo+Bev.

### 3.4 Differences in survival between the two treatments according to treatment response

**Figure 2:**
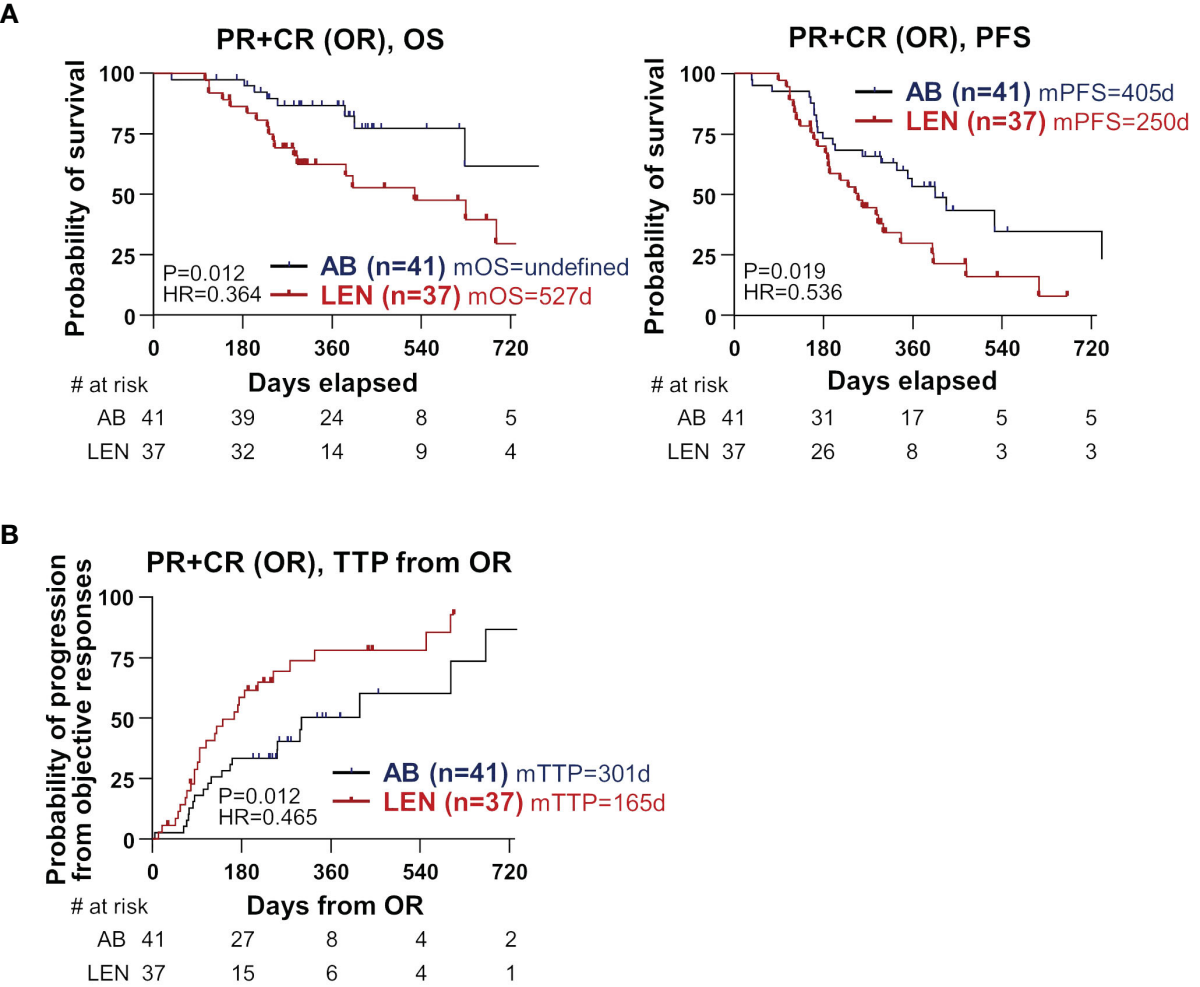
Differences in clinical outcome between the two treatments among the subgroup that achieved objective responses. **(A)** OS and PFS were compared between AB- and LEN-treated patients. **(B)** TTP from the time of OR was compared between the two treatments. PR, partial response; CR, complete response; OR, objective responses; OS, overall survival; PFS, progression-free survival; AB, atezolizumab+bevacizumab; LEN, lenvatinib; m, median; d, days; HR, hazard ratio, TTP, time-to-progression.

We hypothesized that there might be a difference in the OS in patients who achieved OR, because of the better OS in the AB group without superior ORR. We compared OS in patients who achieved OR, and observed significantly better OS in the AB group (n=41) compared to the lenvatinib group (n=37) (median not reached versus 527 days, HR=0.364, P=0.012) (**Figure 2A**, left). Furthermore, PFS was also superior in the AB group compared to the lenvatinib group (median 405 versus 250 days, HR=0.536, P=0.019) (**Figure 2A**, right). Of note, time-to-progression (TTP) from the time point of OR was significantly better in the AB group (median 301 versus 165 days, HR=0.465, P=0.012) (**Figure 2B**). However, there was no difference in OS and PFS among disease-controlled patients (**Supplementary Figure 1A**), as well as in OS among patients with PD (**Supplementary Figure 1B**).

### 3.5 AEs and their association with the survivals

**Table 4** shows the safety profiles of respective treatments. AEs of any grade or serious AEs of grade 3 or more did not differently occur in the two groups. Hand-foot syndrome was only observed in the lenvatinib group (19/141, 13.5%), and thyroiditis was observed more in the AB group (18/141, 12.8% versus 2/141, 1.4%, P=0.001). Variceal bleeding also significantly occurred in the AB group (9/141, 6.4% versus 1/141, 0.6%, P=0.007). However, the chemotherapy was stopped due to AEs more frequently in the lenvatinib group (22/141, 15.6% versus 9/141, 6.4%, P=0.022). Additionally, we have clarified that the median timing of the best responses between the two treatments—70.5 days for lenvatinib and 64 days for AB (P=0.149)—is not significantly different. This indicates that the most common timing for the best responses in both treatments corresponds to the first response evaluation.

**Table 4:** Safety and the duration of chemotherapy of the matched cohort.

	Lenvatinib n=141	AB n=141	P
Adverse events of any grade	64 (45.4)	66 (46.8)	0.905
Hand-foot syndrome	19 (13.5)	0 (0)	<0.001
Diarrhea	7 (5.0)	3 (2.1)	0.334
General weakness, poor oral intake	10 (7.1)	8 (5.7)	0.808
Hypertension	7 (5.0)	3 (2.1)	0.334
Proteinuria	3 (2.1)	5 (3.5)	0.720
Thyroiditis	2 (1.4)	18 (12.8)	0.001
Varix bleeding	1 (0.6)	9 (6.4)	0.007
Hepatic encephalopathy	2 (1.4)	0 (0)	0.478
Decreased liver function	7 (5.0)	16 (11.3)	0.082
Pneumonitis	0 (0)	1 (0.7)	1.000
Serious adverse events	33 (23.4)	27 (19.1)	0.467
Cessation of chemotherapy due to adverse events	22 (15.6)	9 (6.4)	0.022
Median chemotherapy duration, days	102	91	0.907
Median timing of the best responses, days	70.5	64	0.149

AB, Atezolizumab plus Bevacizumab.

Specifically, in the lenvatinib group, older patients (who discontinued treatment) had a significantly higher age than those who continued, indicating a higher susceptibility or lower tolerance to AEs among the elderly. Conversely, age did not play a significant role in treatment discontinuation within the AB group. Additionally, the incidence of general weakness/poor oral intake was markedly higher among patients who stopped lenvatinib due to AEs (22.7%) compared to those who did not (4.2%), highlighting that certain AEs, particularly general weakness and poor oral intake, were critical factors in the decision to discontinue lenvatinib. Other notable AEs such as variceal bleeding, liver function abnormalities, autoimmune side effects, and renal function abnormalities did not significantly impact the decision to discontinue treatment in either group. Thus, older age and a decline in general condition may be more closely associated with treatment discontinuation in lenvatinib compared to AB, though further large-scale studies are needed for confirmation (**Supplementary Table 5**).

We investigated whether the occurrence of AEs causes a difference in survival between the two groups. Among patients with any grade of AE, there was no difference in OS or PFS between the two groups (**Supplementary Figures 2A, B**). Among patients without AEs, there was a tendency for better PFS (P=0.057) observed in the AB group (**Supplementary Figure 2B**). Furthermore, significantly better OS was observed in the AB group (P<0.001) (**Supplementary Figure 2A**). A similar tendency was observed when we divided patients according to serious AEs (**Supplementary Figures 2C, D**).

### 3.6 Impact of residual liver function on survival following the cessation of treatment

**Figure 3:**
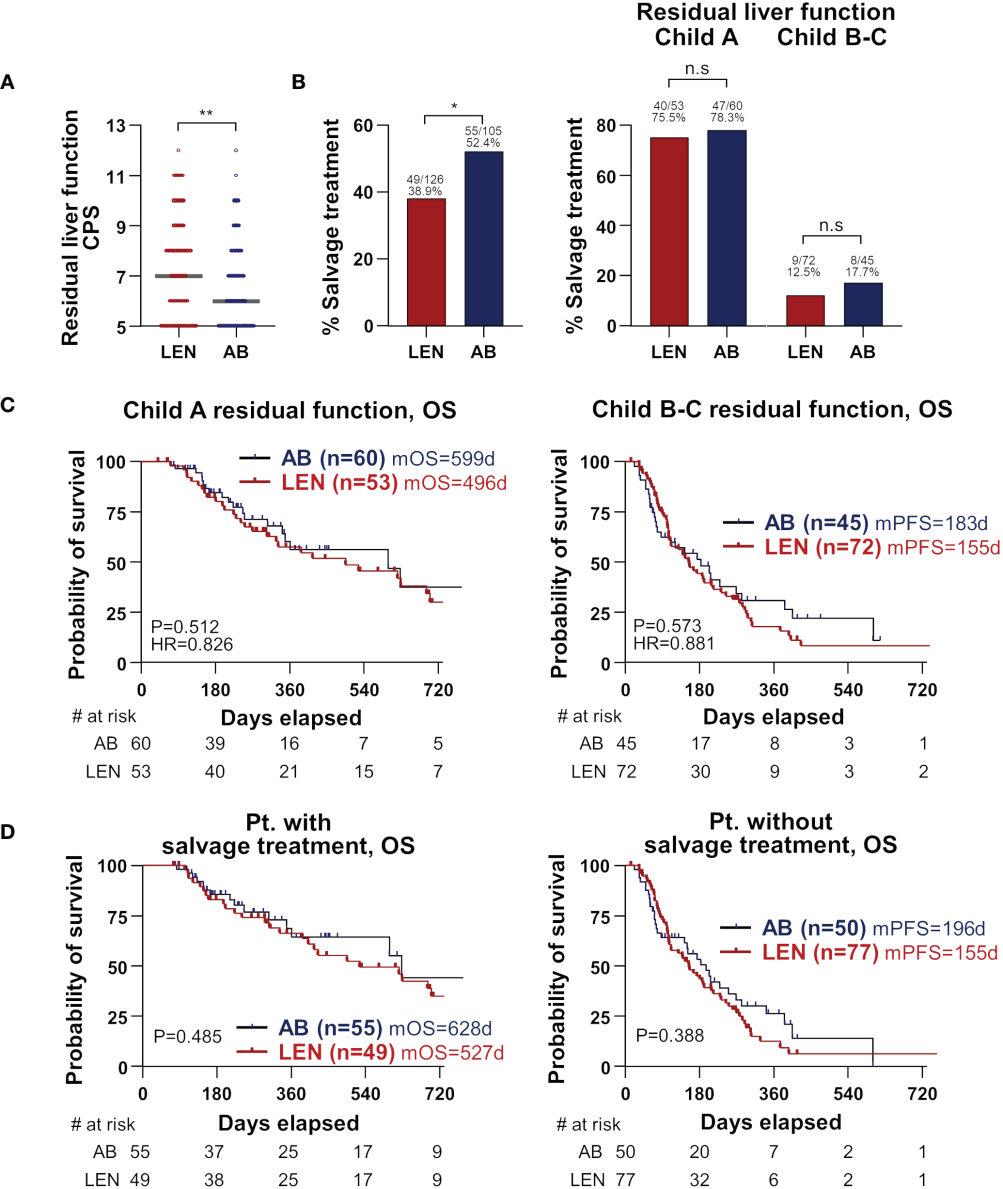
Importance of residual liver function at treatment cessation. **(A)** Residual liver function at treatment cessation is represented by CPS and compared between the two treatments. Median values are presented by grey lines. **(B)** The percentages who received salvage treatments following treatment cessation were compared between the two treatments (left). Patients were divided by Child-Pugh classes, and again the two treatments were compared for the percentages of salvage treatment (right). **(C, D)** Subgroup analyses according to residual liver function **(C)** and salvage treatment **(D)** were performed. OS and PFS were compared between the two treatments. CPS, Child-Pugh score; OS, overall survival; PFS, progression-free survival; AB, atezolizumab+bevacizumab; LEN, lenvatinib; m, median; d, days; HR, hazard ratio; Pt., patients.

We finally hypothesized that residual liver function at the cessation of treatment and subsequent salvage treatment might be associated with the better OS of AB in our cohort. The inclusion criteria for the salvage treatment were as follows; (1) sufficient liver reserve function, classified as Child-Pugh A and B7; (2) ECOG performance status ranging from 0 to 2; and (3) patient consent to undergo salvage treatment. Residual liver function represented by CPS and the frequency and types of salvage treatment are presented in **Supplementary Table 6**. At the time of treatment cessation, the AB group demonstrated superior residual liver function and ECOG performance status. Specifically, median CPS was significantly better in the AB group compared to the lenvatinib group (6 versus 7, P=0.008) (**Figure 3A**). The frequency of salvage treatment was also higher in the AB group (55/105, 52.4% versus 49/126, 38.9%, P=0.048), but there was no difference in the frequency of salvage treatment between the two treatments in each Child-Pugh class group (**Figure 3B**). Furthermore, in patients with the residual function of Child-Pugh A, there was no difference in OS between the two groups, and this result was also observed in the Child-Pugh B-C subgroup (**Figure 3C**). Also, there was no survival difference between the two groups when we performed subgroup analyses according to the salvage treatment or none (**Figure 3D**). There was no significant difference in tumor characteristics, including tumor markers, size, number, portal vein invasion, and extrahepatic spread, between the two treatment groups at the point of salvage treatment initiation (**Supplementary Table 6**). These results suggest that residual liver function and subsequent salvage treatment have an important role in the survival difference between AB and lenvatinib treatments.

## 4 Discussion

In the present study, we conducted a real-world, multi-center study using PSM, which provides a robust comparison between AB and lenvatinib in treating unresectable HCC. AB demonstrated superior OS compared to lenvatinib, without a significant difference in PFS or ORR. This underscores that PFS alone may not reflect the true benefit of a treatment, particularly in the context of immunotherapy for HCC, which is in line with the previous report (16). Our research highlights other factors influencing OS benefits with AB treatment, such as the importance of residual liver function post-treatment and the role of salvage treatments. Interestingly, in patients who achieved OR by respective treatments, OS, PFS, and TTP were better in the AB group, suggesting durable response can be achieved by this regimen compared to lenvatinib. Of note, our study highlights that residual liver function following the cessation of respective treatments, as measured by the CPS, is a critical determinant of OS, and the difference in residual liver function might be associated with the different frequency of subsequent salvage treatment and OS between two groups. Treatment discontinuation due to AEs was more frequent in the lenvatinib group, but the incidence of serious AEs was not different between the two treatments. The superiority of AB in OS was diminished in patients who underwent serious AEs, suggesting the prediction, monitoring, and management of AEs are also important in both treatments. Subgroup analysis showed that the subgroups of patients with high tumor burden or preserved liver function had significant survival benefits from AB.

Studies comparing these two regimens have conflicting results, although most studies used matched cohorts using PSM or inverse probability of treatment weighting (3–6, 8, 9). Some studies showed that AB has superior OS (4, 5) or PFS (3–5) compared to lenvatinib. Other reports showed that these two treatments have comparable OS and PFS (8, 9). However, another study demonstrated that lenvatinib was associated with longer OS and PFS (6). Two recent meta-analyses also reported different results (17, 18). A study analysing 6,628 patients from 8 studies showed that there was no difference in OS and PFS between two treatments (17), but another study analysing 3,690 patients from 8 studies showed longer PFS of AB treatment (17). These results might be related to the characteristics of the cohort, which can be baseline or post-treatment events such as AEs, residual liver function, and salvage treatments. Our study particularly focused on the analysis of factors associated with the difference in clinical outcomes between the two treatments.

One of the important findings is that we first identified that residual liver function after treatment cessation is significantly associated with the patient survival in both treatments, which can be associated with the difference of clinical outcomes between two treatments. We showed that this residual liver function affected the difference in OS between the two regimens. Patients who have better residual liver function after cessation of the primary treatments might have better survival because of eligibility for further treatment. Moreover, there were no survival differences between the two treatments if residual liver function was adjusted for. This underscores the need to maintain liver function during systemic therapies. Previous studies reported that AB caused worsening of ALBI score 3 weeks after treatment, but tended to be maintained thereafter (19–21). Other reports showed that lenvatinib treatment was associated with worsening of ALBI score at 2 and 4 weeks of treatment (22). A recent comparative study confirmed that this difference of dynamic changes within 6 weeks after respective treatments (3). However, these studies were focused on the short-term changes in liver function following each treatment, which would not be directly related to preserved liver function at the time of treatment cessation that is necessary for subsequent treatment. Rather, our study directly compared residual liver function at the cessation of each treatment regardless of the time point and found that CPS was significantly better in the AB group, which might be related to better OS.

Our second critical finding is that subsequent opportunity to receive salvage treatment is related to the survival difference between the two treatments. The AB group more frequently received salvage treatment, which resulted in better OS than the lenvatinib group. In addition, there was no survival difference between the two treatments if the salvage treatment was adjusted. Subsequent treatment was significantly associated with better survival in lenvatinib-treated HCC patients (23). In the AB treatment, salvage treatment was also analysed and its importance was also discussed in a recent report, but a direct comparison of salvage treatment between lenvatinib and AB treatments, or its prognostic impact were not examined (5). The frequency of salvage treatment can be heterogeneous among different cohorts; for example, post-progression treatment was performed in 77.6% following AB treatment in the Japanese cohort (5), but it was 52.4% in our cohort. Therefore, subsequent treatments following cessation might be associated with the heterogeneous survival results from previous studies. These findings imply that future comparative studies should consider residual liver function and subsequent treatment after the treatment cessation.

The third notable finding is that even in the subgroup that achieved OR, AB showed better OS and PFS. Furthermore, TTP from the time of OR was longer in AB than lenvatinib, suggesting that AB treatment could have long-term anti-tumor effects. Immunotherapies in HCC can induce durable responses which can result in prolonged survival (24, 25). The CheckMate459 trial also showed that nivolumab was more durable than sorafenib in terms of disease control (26). This relatively long-term effect of immunotherapies can be explained by the augmentation of tumor-specific memory responses which mainly recognize cancer cells (27). Moreover, our study also showed that treatment discontinuation due to AEs was significantly higher in the lenvatinib treatment, which can influence the durability of the treatment responses. Thus, the long-term beneficial effect of AB treatment, compared to lenvatinib treatment, should be investigated in future translational and clinical research.

We also performed detailed subgroup analyses using a matched cohort to identify which treatment would be beneficial in terms of OS and PFS in the respective subgroups. A recent experimental report suggested that the limited role of anti-PD-1 treatment in NASH-related HCC might be due to the pathologic CD8+PD-1+ T cells (28). In the latest results from the IMbrave150 study, treatment with AB showed better OS and PFS in patients with viral causes like HBV and HCV compared to sorafenib (29). Moreover, a recent network meta-analysis revealed that AB treatment could be beneficial in terms of survival in the subgroup of viral etiology (30). Our study also confirmed that AB treatment might have a comparative benefit in OS in viral etiology, but not in non-viral etiology. In addition to the etiology, we first observed that patients with high tumor burden reflected by tumor markers, size, number of intrahepatic tumors, and PVI had a benefit in OS and PFS from AB treatment than lenvatinib treatment. In addition, tumor size, tumor markers, or PVI were not factors associated with poor clinical outcomes in the AB subgroup, unlike the lenvatinib subgroup. These findings could support the treatment decision between the two treatments, although more data need to be accumulated in future studies.

Despite the retrospective design, our multi-center real-world study is the first to suggest that residual liver function and subsequent salvage treatments are the major determinants of clinical outcomes in HCC patients treated with AB and lenvatinib. These factors might be associated with the conflicting results of previous comparative studies, and this point should be considered in future studies. Importantly, cautious monitoring and management to maintain liver function during those treatments would consequently improve patient outcomes in advanced HCC.

## Data availability statement

The original contributions presented in the study are included in the article/**Supplementary Material**. Further inquiries can be directed to the corresponding author.

## Ethics statement

The studies involving humans were approved by Ethical approval for this research was granted by the Institutional Review Board of the Catholic University of Korea (approved number: XC23RADI0081). The studies were conducted in accordance with the local legislation and institutional requirements. The ethics committee/institutional review board waived the requirement of written informed consent for participation from the participants or the participants’ legal guardians/next of kin because this study was a retrospective study.

## Author contributions

JH: Writing – original draft, Writing – review & editing, Conceptualization, Data curation, Formal analysis, Investigation, Methodology, Resources, Visualization. PS: Writing – original draft, Writing – review & editing, Funding acquisition, Resources, Supervision, Validation. JY: Resources, Writing – review & editing. HC: Resources, Writing – review & editing. SL: Resources, Writing – review & editing. HY: Resources, Writing – review & editing. JKim: Resources, Writing – review & editing. HN: Resources, Writing – review & editing. HL: Resources, Writing – review & editing. HK: Resources, Writing – review & editing. SL: Resources, Writing – review & editing. DS: Resources, Writing – review & editing. MS: Resources, Writing – review & editing. JKwon: Resources, Writing – review & editing. CK: Resources, Writing – review & editing. SB: Resources, Writing – review & editing. JJ: Resources, Writing – review & editing. JC: Resources, Writing – review & editing. SY: Resources, Writing – review & editing.
