# Supplementary figures and images for: Differential liver function at cessation of atezolizumab-bevacizumab versus lenvatinib in HCC: a multicenter, propensity-score matched comparative study
Source: Front Oncol. 2024 Feb 28;14:1372007. doi: 10.3389/fonc.2024.1372007 (PMC10933027; doi:10.3389/fonc.2024.1372007)

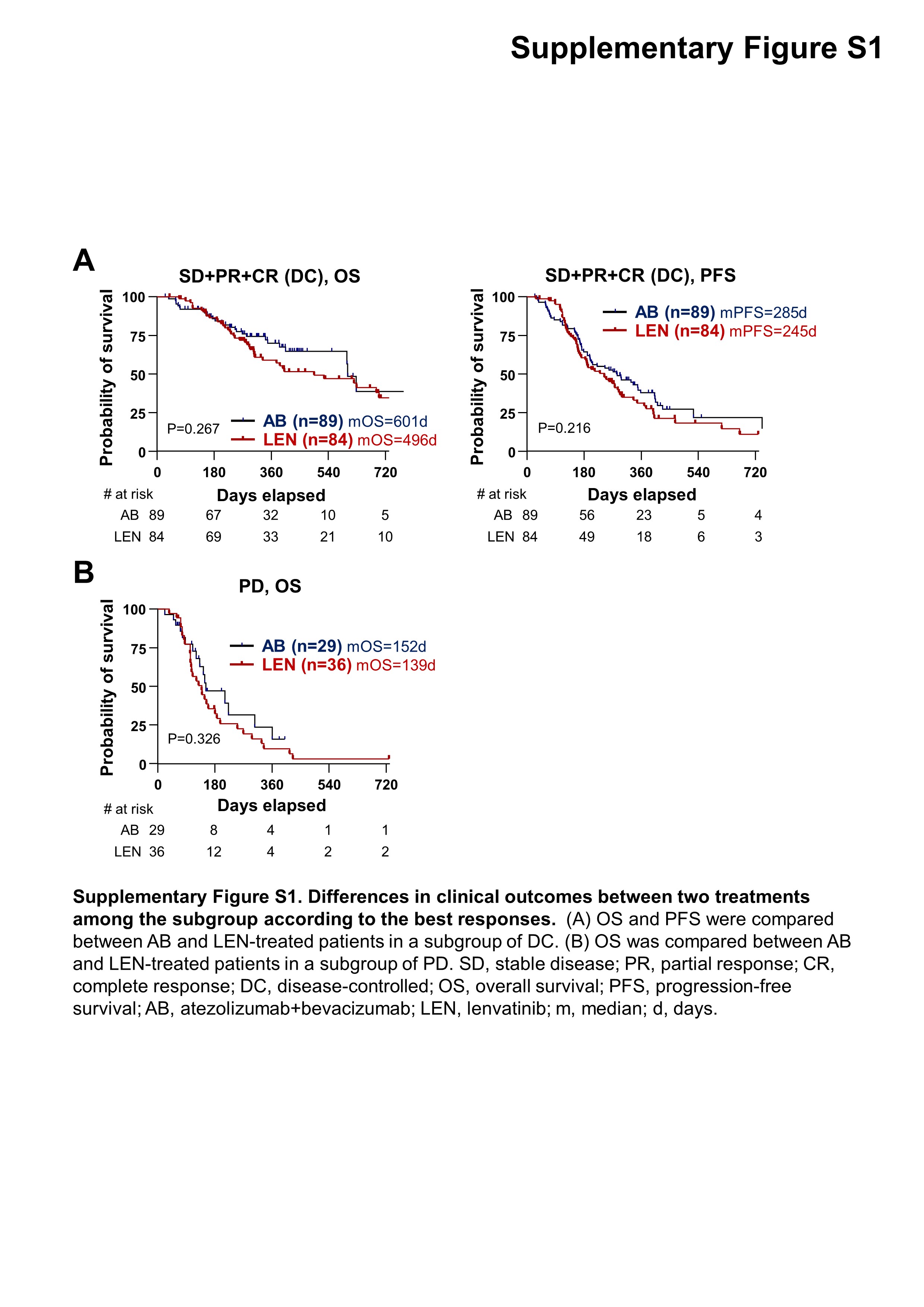

Supplement: Supplementary file 1 [file Image_1.jpeg]

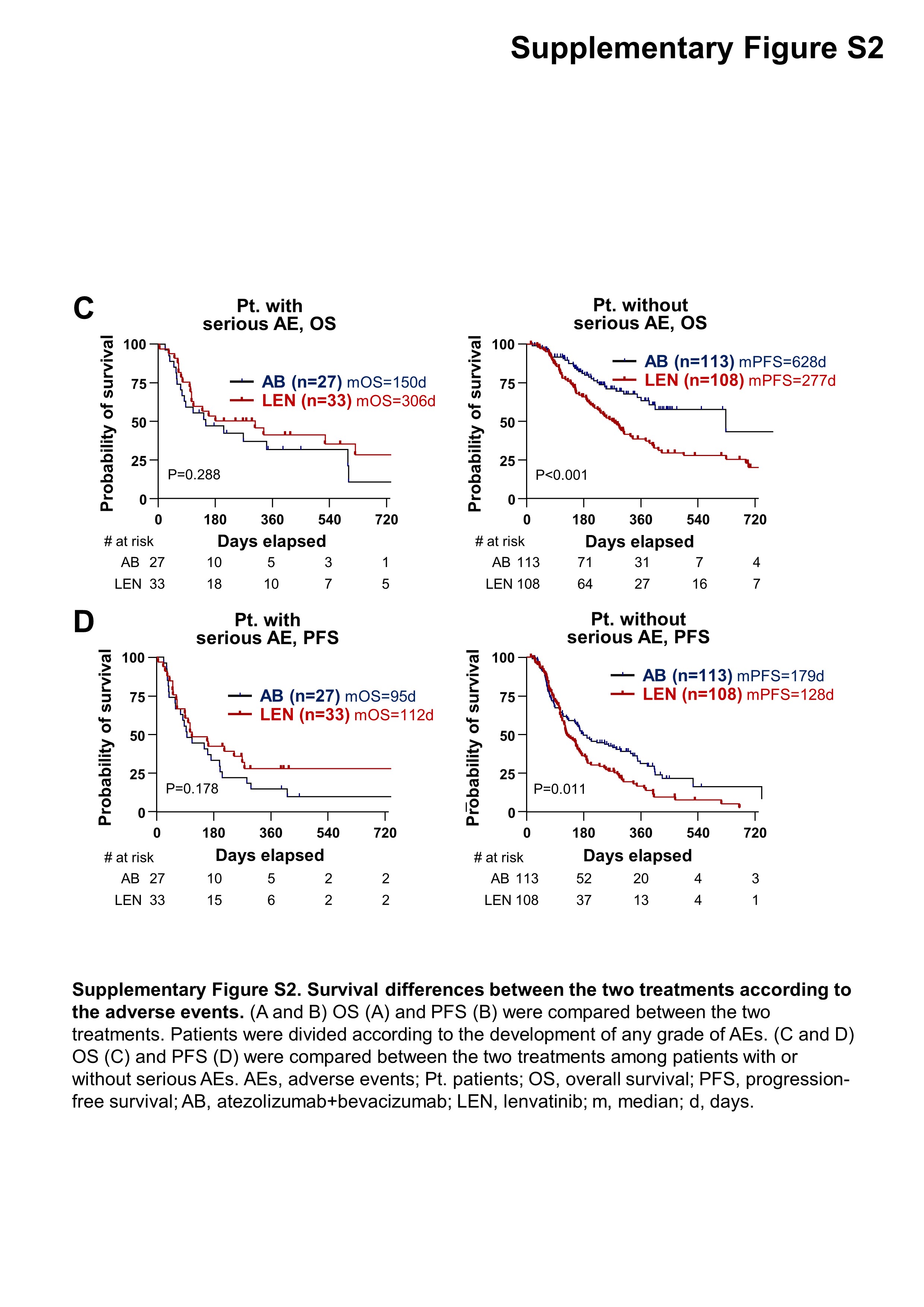

Supplement: Supplementary file 2 [file Image_2.jpeg]
