# Supplementary material for: Differential liver function at cessation of atezolizumab-bevacizumab versus lenvatinib in HCC: a multicenter, propensity-score matched comparative study
Source: Front Oncol. 2024 Feb 28;14:1372007. doi: 10.3389/fonc.2024.1372007 (PMC10933027; doi:10.3389/fonc.2024.1372007)
Supplement: Supplementary file 3 [file Table_1.docx]

**Supplementary Table S1.** Subgroup analyses comparing two treatments in terms of overall survival in the matched cohort

|  | HR* | P |
| --- | --- | --- |
| Age>65 | 0.578 (0.38-0.88) | 0.0126 |
| Treatment naïve | 0.597 (0.34-1.06) | 0.079 |
| Viral etiology | 0.568 (0.39-0.83) | 0.005 |
| AST≤40 | 0.581 (0.31-1.08) | 0.0862 |
| ALBI grade 1 | 0.371 (0.19-0.70) | 0.0059 |
| Platelet>150 | 0.669 (0.42-1.05) | 0.0844 |
| AFP>1000 | 0.514 (0.30-0.86) | 0.0173 |
| PIVKA-II>1000 | 0.609 (0.39-0.93) | 0.024 |
| ECOG 0 | 0.650 (0.42-0.98) | 0.0465 |
| Child-Pugh 5A | 0.516 (0.31-0.85) | 0.0147 |
| Largest intrahepatic tumor>5cm | 0.520 (0.34-0.78) | 0.0016 |
| Multiple intrahepatic tumor | 0.521 (0.36-0.74) | 0.0005 |
| Portal vein invasion | 0.536 (0.35-0.81) | 0.0037 |
| Extrahepatic metastasis | 0.657 (0.42-1.01) | 0.1617 |

HR, Hazard Ratio; AST, Aspartate Aminotransferase; ALBI, albumin-bilirubin; AFP, Alpha-fetoprotein; PIVKA-II, Protein Induced by Vitamin K Absence or Antagonist-II; ECOG, Eastern Cooperative Oncology Group.

*HR was calculated by the log-rank tests, and presented as atezolizumab+bevacizumab versus lenvatinib. HR < 1 = favor for atezolizumab+bevacizumab

**Supplementary Table S2.** Subgroup analyses comparing two treatments in terms of progression-free survival in the matched cohort

|  | HR* | P |
| --- | --- | --- |
| Age>65 | 0.768 (0.54-1.08) | 0.1293 |
| Treatment naïve | 0.753 (0.44-1.28) | 0.2893 |
| Viral etiology | 0.830 (0.61-1.14) | 0.2402 |
| AST≤40 | 0.749 (0.48-1.15) | 0.1803 |
| ALBI grade 1 | 0.635 (0.40-1.00) | 0.0465 |
| Platelet>150 | 0.685 (0.47-0.98) | 0.0372 |
| AFP>1000 | 0.675 (0.43-1.05) | 0.0751 |
| PIVKA-II>1000 | 0.692 (0.48-0.99) | 0.0377 |
| ECOG 0 | 0.752 (0.53-1.05) | 0.09 |
| Child-Pugh 5A | 0.797 (0.55-1.15) | 0.2256 |
| Largest intrahepatic tumor>5cm | 0.705 (0.49-1.00) | 0.0422 |
| Multiple intrahepatic tumor | 0.710 (0.52-0.96) | 0.0269 |
| Portal vein invasion | 0.675 (0.46-0.98) | 0.0365 |
| Extrahepatic metastasis | 0.911 (0.65-1.27) | 0.5807 |

HR, Hazard Ratio; AST, Aspartate Aminotransferase; ALBI, albumin-bilirubin; AFP, Alpha-fetoprotein; PIVKA-II, Protein Induced by Vitamin K Absence or Antagonist-II; ECOG, Eastern Cooperative Oncology Group.

*HR was calculated by the log-rank tests, and presented as atezolizumab+bevacizumab versus lenvatinib. HR < 1 = favor for atezolizumab+bevacizumab

**Supplementary Table S3.** Multivariate Cox-regression analyses* of factors associated with progression-free survival in the matched cohort

|  | Total (n=282) | | Lenvatinib (n=141) | | AB (n=141) | |
| --- | --- | --- | --- | --- | --- | --- |
|  | HR | P | HR | P | HR | P |
| AST | 1.003 (1.00-1.01) | 0.0008 | 1.004 (1.00-1.01) | 0.0121 | 1.003 (1.00-1.01) | 0.0509 |
| Albumin | not included* | | not included | | 0.441 (0.27-073) | 0.0016 |
| AFP | 1 (1.00-1.00) | 0.1243 | 1.00 (1.00-1.00) | 0.232 | not included | |
| Ascites | not included | | not included | | 1.701 (1.02-2.84) | 0.042 |
| ECOG | 1.652 (1.26-2.17) | 0.0003 | not included | | 1.916 (1.32-2.78) | 0.0006 |
| Child-Pugh score | 1.408 (1.19-1.67) | < .0001 | 1.494 (1.20-1.87) | 0.0004 | not included | |
| Multiple intrahepatic tumor | 1.422 (1.03-1.96) | 0.0305 | 1.926 (1.23-3.02) | 0.0043 | not included | |
| mUICC stage | 1.334 (1.13-1.58) | 0.0008 | 1.343 (1.07-1.69) | 0.0112 | 1.392 (1.07-1.81) | 0.0132 |

AB, Atezolizumab plus Bevacizumab; HR, hazard ratio; AST, Aspartate Aminotransferase; AFP, Alpha-fetoprotein; ECOG, Eastern Cooperative Oncology Group; mUICC, Modified Union for International Cancer Control.

* Only factors with P<0.02 in univariate analyses were included for multivariate analyses.

**Supplementary Table S4.** Multivariate logistic-regression analyses of factors associated with objective responses in the matched cohort

|  | Total (n=282) | | Lenvatinib (n=141) | | AB (n=141) | |
| --- | --- | --- | --- | --- | --- | --- |
|  | HR | P | HR | P | HR | P |
| Male gender |  |  |  |  | 3.21 (0.99-14.50) | 0.078 |
| AST | 0.99 (0.99-1.00) | 0.016 | 0.99 (0.98-1.00) | 0.172 | 0.99 (0.98-1.00) | 0.05 |
| INR | not included | | 0.02 (0.00-1.48) | 0.09 |  | |
| ECOG | 0.43 (0.23-0.76) | 0.006 | 0.37 (0.13-0.96) | 0.053 | 0.52 (0.23-1.09) | 0.1 |
| Multiple intrahepatic tumor | not included | | 0.52 (0.22-1.26) | 0.144 |  |  |

AB, Atezolizumab plus Bevacizumab; HR, Hazard Ratio; AST, Aspartate Aminotransferase; INR, International Normalized Ratio; ECOG, Eastern Cooperative Oncology Group.

**Supplementary Table S5.** Comparison between patients according to the cessation of chemotherapy due to adverse events among each treatment group

|  | AB | |  | Lenvatinib | |  |
| --- | --- | --- | --- | --- | --- | --- |
| Cessation of chemotherapy due to AEs | No | Yes |  | No | Yes |  |
|  | n=132 | n=9 | P | n=119 | n=22 | P |
| Age | 63.5 ± 11.1 | 63.7 ± 13.0 | 0.975 | 62.4 ± 11.1 | 71.3 ± 13.3 | 0.001 |
| Serious AEs | 18 (13.6%) | 9 (100.0%) | < 0.001 | 11 (9.2%) | 22 (100.0%) | < 0.001 |
| General weakness/Poor oral intake | 8 (6.1%) | 0 (0.0%) | 0.987 | 5 (4.2%) | 5 (22.7%) | 0.008 |
| Variceal bleeding | 9 (6.8%) | 0 (0.0%) | 0.916 | 0 (0.0%) | 0 (0.0%) | 1 |
| Liver function abnormality | 16 (12.1%) | 0 (0.0%) | 0.571 | 5 (4.2%) | 2 (9.1%) | 0.663 |
| Autoimmune side effects | 28 (21.2%) | 4 (44.4%) | 0.231 | 0 (0.0%) | 0 (0.0%) | 1 |
| Renal function abnormality | 5 (3.8%) | 0 (0.0%) | 1 | 2 (1.7%) | 1 (4.5%) | 0.959 |

Data are given as n (%). AB, Atezolizumab plus Bevacizumab; AEs, adverse events.

**Supplementary Table S6.** Characteristics at the cessation of each treatment and salvage treatments in the matched cohort

|  | Lenvatinib n=126 | AB n=105 | P |
| --- | --- | --- | --- |
| Residual liver function, CPS | 7.3 ± 2.0 | 6.6 ± 1.7 | 0.004 |
| ECOG |  |  | 0.017 |
| 0 | 35 (28.0) | 39 (37.1) |  |
| 1 | 33 (26.4) | 39 (37.1) |  |
| 2 | 36 (28.8) | 19 (18.1) |  |
| 3 | 21 (16.8) | 8 (7.6) |  |
| Refuse for the salvage treatment | 8 (6.3) | 3 (2.9) | 0.352 |
| Salvage treatment | 49 (38.9) | 55 (52.4) | 0.048 |
| TACE | 11 (22.4) | 6 (10.9) |  |
| HAIC | 5 (10.2) | 11 (20.0) |  |
| Sorafenib | 21 (42.9) | 24 (43.6) |  |
| Radiotherapy | 4 (8.2) | 12 (21.8) |  |
| Others | 6 (12.2) | 2 (3.6) |  |
| Tumor status at salvage treatment |  |  |  |
| AFP, ng/mL | 13844.4 ± 30650.2 | 7043.7 ± 15808.5 | 0.167 |
| PIVKA-II, mAU/mL | 18617.5 ± 51476.0 | 17868.8 ± 43722.7 | 0.936 |
| Largest intrahepatic tumor size, cm | 30 (61.2) | 40 (72.7) | 0.588 |
| Multiple intrahepatic tumor | 30 (61.2) | 40 (72.7) | 0.299 |
| Portal vein invasion | 16 (32.7) | 24 (43.6) | 0.343 |
| Extrahepatic spread | 29 (59.2) | 28 (50.9) | 0.516 |

AB, Atezolizumab plus Bevacizumab; CPS, Child-Pugh Score; TACE, Transarterial Chemoembolization; HAIC, Hepatic Artery Infusion Chemotherapy; AFP, alpha-fetoprotein; PIVKA-II, protein induced by vitamine K antagonist.
